# Survey of healthcare providers’ testing practices for vulvovaginal candidiasis and treatment outcomes–United States, 2021

**DOI:** 10.1371/journal.pone.0278630

**Published:** 2022-12-30

**Authors:** Kaitlin Benedict, Ravan Moret, Noelle Angelique M. Molinari, Brendan R. Jackson

**Affiliations:** Division of Foodborne, Waterborne and Environmental Diseases, National Center for Emerging and Zoonotic Infectious Diseases, Centers for Disease Control and Prevention, Atlanta, Georgia, United States of America; Tulane University School of Public Health and Tropical Medicine, UNITED STATES

## Abstract

Vulvovaginal candidiasis (VVC) is a common infection, and high-quality studies report that misdiagnosis is frequent, with diagnostic testing needed to distinguish it from other causes of vaginitis and avoid inappropriate empiric treatment. However, few recent studies have evaluated U.S. healthcare providers’ testing practices for VVC in detail. We evaluated healthcare providers’ self-reported testing practices for VVC and treatment outcomes as part of a nationwide online survey in order to identify potential opportunities for improving VVC testing and treatment in the United States. Among 1,503 providers surveyed, 21.3% reported “always” (7.4%) or “usually” (13.9%) ordering diagnostic testing for patients with suspected VVC; this proportion was higher among gynecologists (36.0%) compared with family practitioners (17.8%) and internists (15.8%). Most providers (91.2%) reported that patients’ VVC “always” (6.4%) or “usually” (84.9%) responds to initial treatment. Whether the symptom resolution reported in this survey was truly related to VVC is unclear given high rates of misdiagnosis and known widespread empiric prescribing. With only about one-in-five providers reporting usually or always performing diagnostic testing for VVC despite guidelines recommending universal use, research is needed to address barriers to proper testing.

## Introduction

Vaginitis is the most common gynecologic patient complaint in primary care settings [1,2]. Vulvovaginal candidiasis (VVC) accounts for approximately one-third of vaginitis cases and is characterized by symptoms such as vaginal itching, burning, pain, and discharge. Over half of women are affected by VVC during their lifetime [3], with >5% experiencing recurrent episodes [4], resulting in substantial morbidity in terms of both physical and mental health.

Due to the clinical similarities between VVC and other types of vaginitis, diagnosis based on signs and symptoms alone is unreliable [1,2]. Point-of-care testing (wet mount microscopy, potassium hydroxide [KOH] test, or vaginal pH test) or laboratory-based testing (culture, Gram stain, PCR) can improve the diagnosis of VVC [2,5]. Specifically, wet mount microscopy is recommended for all women with signs and symptoms of VVC, followed by culture if the wet mount results are negative [5]. However, wet mount microscopy appears to be uncommonly performed, in only 17% of women with vaginitis [6]. Lack of diagnostic testing often results in inappropriate empiric treatment (raising concerns for antimicrobial resistance) and prolonged symptoms [6,7]. Typically, VVC can be successfully treated with a short course of topical or oral antifungal medication, although a longer treatment course is usually needed for severe or recurrent infections [5].

Few recent studies have evaluated U.S. healthcare providers’ (HCP) testing practices for VVC in detail. Therefore, we examined HCPs’ self-reported testing practices and treatment outcomes in order to identify potential opportunities to improve testing and treatment for VVC.

## Methods

A web-based survey of primary care physicians, obstetricians and gynecologists (OB/GYNs), pediatricians, nurse practitioners, and physician assistants was conducted in spring 2021. This survey (DocStyles) was commissioned by Porter Novelli Public Services, a public relations firm, and conducted by SERMO, a global market research company. The survey aimed to provide insight into HCPs’ attitudes and practices about various health conditions and assess their use of health information sources. SERMO sampled its active panel members to reach quotas of 1,000 primary care physicians, 250 OB/GYNs, 250 pediatricians, and 250 nurse practitioners/physician assistants. Respondents were screened to include only those actively seeing patients in the United States and practicing for at least 3 years. Porter Novelli developed the survey instrument with guidance from federal public health agencies and other clients. Two questions were asked of all HCPs except pediatricians. First, “For patients in whom you suspect vulvovaginal candidiasis, do you usually order diagnostic testing, or do you treat empirically (without diagnostic testing)?” Response options were: “Always order diagnostic testing,” “Usually order diagnostic testing,” “A mix of both,” “Usually treat empirically," and “I don’t see patients with VVC.” Second, among those who saw patients with VVC, HCPs were asked “Among your patients with vulvovaginal candidiasis (VVC), how often does their VVC respond to initial treatment?” Response options were: “Always,” “Usually,” “Sometimes,” “Rarely,” and “Never.” Due to small cell counts in cross-tabulations, responses to both questions were dichotomized.

Descriptive and bivariate analyses were used to assess HCP features (e.g. age, sex) associated with VVC testing practices and treatment outcomes, using t-tests for continuous variables and chi-squared tests for categorical variables. Multivariable logistic regression estimated adjusted odds ratios (aORs) and 95% confidence intervals (95% CI) for factors associated with testing practices, using augmented backward selection of variable for inclusion. The final model was bootstrapped to adjust standard errors for multiplicity using 500 replications. Multivariable analysis was not appropriate for evaluating factors associated with treatment outcomes due to the small number of HCPs who reported poor treatment outcomes; the association between testing practices and treatment outcomes was examined using bivariate analyses and chi-squared tests for association. Analyses were conducted using SAS (version 9.4; SAS Institute).

CDC licensed the data used in this analysis from Porter Novelli. Porter Novelli and Ipsos are not subject to CDC Institutional Board review; however, they adhere to all professional standards and codes of conduct set forth by the ESOMAR Code of Conduct (https://esomar.org/code-and-guidelines/icc-esomar-code) and the Insights Association (https://www.insightsassociation.org/Resources/Codes-of-Standards). Survey respondents were informed that their answers were being used for market research and they could exit the survey at any time. No personal identifiers were included in the data file provided to CDC.

## Results

Of 2,675 HCPs surveyed, 1,753 (65.5%) completed the survey, 1,503 were queried about VVC practices, and 168 (11.2%) were excluded from further analyses because they did not see patients with VVC. Of the remaining 1,335 HCPs, 34.5% were family practitioners, 32.7% were internists, and 18.5% were OB/GYNs (Table 1). Most HCPs (64.0%) practiced in a group outpatient practice or clinic, and the mean number of years in practice was 17.0.

**Table 1:** Healthcare providers’ self-reported testing practices for vulvovaginal candidiasis (VVC) and treatment outcomes by provider characteristic and practice characteristic–Porter Novelli Spring DocStyles Survey, United States, 2021.

		Question 1: testing			Question 2: treatment		
	Total	Always/usually order diagnostic testing (n = 284)	Always/usually treat empirically^a^ (n = 1,051)		Always/usually respond to initial treatment (n = 1,218)	Sometimes/rarely/ never respond to initial treatment (n = 117)	
	n (column %)	n (row %)	n (row %)	p-value	n (row %)	n (row %)	p-value
**Provider characteristics**							
Age in years, mean (SD)	47 (11.5)	46.2 (11.4)	47.2 (11.5)	0.959	47.1 (11.6)	45.4 (10.3)	0.101
Gender							
Male	769 (57.6)	152 (19.8)	617 (80.2)	0.118	700 (91.0)	69 (9.0)	0.753
Female	566 (42.4)	132 (23.3)	434 (76.7)		518 (91.5)	48 (8.5)	
Race/ethnicity							
White/non-Hispanic	875 (65.5)	187 (21.4)	688 (78.6)	0.622	805 (92.0)	70 (8.0)	0.624
Black/non-Hispanic	42 (3.1)	7 (16.7)	35 (83.3)		38 (90.5)	4 (9.5)	
Asian/non-Hispanic	276 (20.7)	64 (23.2)	212 (76.8)		247 (89.5)	29 (10.5)	
Multiple or other race/non-Hispanic	76 (5.7)	12 (15.8)	64 (84.2)		67 (88.2)	9 (11.8)	
Hispanic	66 (4.9)	14 (21.2)	52 (78.8)		61 (92.4)	5 (7.6)	
Specialty							
Family practitioner	461 (34.5)	82 (17.8)	379 (82.2)	<0.0001	426 (92.4)	35 (7.6)	0.028
Internist	437 (32.7)	69 (15.8)	368 (84.2)		383 (87.6)	54 (12.4)	
Obstetrician/gynecologist	247 (18.5)	89 (36.0)	158 (64.0)		232 (93.9)	15 (6.1)	
Nurse practitioner or physician assistant	190 (14.2)	44 (23.2)	146 (76.8)		177 (92.1)	13 (7.9)	
Teaching hospital privileges	620 (46.4)	137 (22.1)	483 (77.9)	0.494	563 (90.8)	57 (9.2)	0.606
Years in practice, mean (SD)	17 (9.5)	16.4 (9.4)	16.8 (9.6)	0.620	16.7 (9.7)	16.3 (8.2)	0.027
Number of patients per week, mean (SD)	103 (65.6)	101.1 (65.7)	103.1 (65.5)	0.951	100.3 (61.7)	127.2 (94.2)	<0.0001
See pediatric patients	959 (71.8)	233 (24.3)	726 (75.7)	<0.0001	885 (92.3)	74 (7.7)	0.035
Approximate patient household income, mean (SD)^b^	3 (1.0)	2.0 (1.0)	2.0 (1.0)	0.216	2.0 (1.0)	2.3 (1.0)	0.630
**Practice characteristics**							
Setting				0.789			0.139
Individual outpatient practice	249 (18.7)	49 (19.7)	200 (80.3)		221 (88.8)	28 (11.2)	
Group outpatient practice or clinic	854 (64.0)	185 (21.7)	669 (78.3)		789 (92.4)	65 (7.6)	
Inpatient practice/hospital	232 (17.4)	50 (21.6)	182 (78.5)		208 (89.7)	24 (10.3)	
Number of providers in practice, mean (SD)	25 (78.7)	21.8 (53.9)	25.8 (84.2)	<0.0001	23.6 (71.0)	38.8 (134.9)	<0.0001
Region				0.011			0.711
Midwest	301 (22.5)	82 (27.2)	219 (72.8)		271 (90.0)	30 (10.0)	
Northeast	298 (22.3)	53 (17.8)	245 (82.2)		276 (92.6)	22 (7.4)	
South	455 (34.1)	84 (18.5)	371 (81.5)		416 (91.4)	39 (8.6)	
West	281 (21.0)	65 (23.1)	216 (76.9)		255 (90.8)	26 (9.3)	
Metropolitan status				0.374			0.086
Urban	476 (35.7)	107 (22.5)	369 (75.5)		424 (89.1)	52 (10.9)	
Suburban	708 (53.0)	151 (21.3)	557 (78.7)		652 (92.1)	56 (7.9)	
Rural	151 (11.3)	26 (17.2)	125 (82.8)		142 (94.0)	9 (6.0)	

^a^ or a mix of both diagnostic testing and empiric treatment.

^b^ a categorical variable where 1: <$25,000, 2: $25,000-$49,000, 3: $50,000-$99,999, 4: $100,000-$249,999, 5: ≥$250,000.

SD = standard deviation.

Total, 21.3% of HCPs reported “always” (7.4%) or “usually” (13.9%) ordering diagnostic testing for patients with suspected VVC; this proportion was highest among OB/GYNs (36.0%) and lowest among internists (15.8%) (p<0.0001). Most (91.2%) HCPs reported that VVC “always” (6.4%) or “usually” (84.9%) responds to initial treatment; this proportion was highest among OB/GYNs (93.9%) and lowest among internists (87.6%) (p = 0.028). A higher mean number of years in practice (16.7 vs. 16.3, p = 0.027) and a lower number of patients per week (100.3 vs. 127.2, p<0.0001) were also associated with seeing patients with VVC who “always” or “usually” respond to initial treatment.

Treatment success was not significantly associated with diagnostic testing frequency; 90.5% of HCPs who “always” or “usually” order testing for VVC (vs. 91.4% of HCPs who treat empirically, p = 0.621) said that their patients with VVC “always” or “usually” respond to initial treatment.

On multivariable analysis, the odds of ordering diagnostic testing were higher among OB/GYNs (aOR 1.49, 95% CI 1.10–2.02) compared with nurse practitioners or physician assistants and in the Midwest (aOR 1.46, 95% CI 1.08–1.99) compared with the Northeast (Table 2). Providers who saw pediatric patients had lower odds of ordering diagnostic testing (aOR 0.59, 95% CI 0.43, 0.80) compared with those who did not see pediatric patients.

**Table 2:** Multivariable analysis of factors associated with “always” or “usually” ordering diagnostic testing for vulvovaginal candidiasis (VVC)–Porter Novelli Spring DocStyles Survey, United States, 2021.

	aOR	95% CI	Adjusted p-value
**Provider characteristics**			
Gender			
Male	reference		
Female	0.96	0.77–1.17	0.67
Race/ethnicity			
White/non-Hispanic	reference		
Black/non-Hispanic	0.80	0.41–1.58	0.52
Asian/non-Hispanic	1.10	0.86–1.42	0.43
Multiple or other race/non-Hispanic	1.13	0.69–1.84	0.61
Hispanic	0.81	0.47–1.40	0.43
Specialty			
Family practitioner	0.74	0.51–1.07	0.08
Internist	0.84	0.57–1.23	0.38
Obstetrician/gynecologist	1.49	1.10–2.02	0.02
Nurse practitioner or physician assistant	reference		
See pediatric patients	0.59	0.43–0.80	0.002
Number of patients per week	1.00	0.99–1.00	0.54
**Practice characteristics**			
Number of providers in practice	1.00	1.00–1.01	0.09
Region			
Northeast	reference		
Midwest	1.46	1.08–1.99	0.02
South	0.99	0.73–1.36	0.97
West	1.23	0.89–1.69	0.23
Metropolitan status			
Suburban	reference		
Urban	0.83	0.57–1.20	0.31
Rural	1.03	0.84–1.27	0.78

## Discussion

In this nationwide survey of HCPs, most (nearly 80%) reported not routinely ordering diagnostic testing for VVC, which is inconsistent with recommendations. However, despite the known inadequacy of empiric diagnosis, HCPs also reported high rates of treatment success, which may be subject to various biases.

The low rates of diagnostic testing reported in this survey are consistent with previous studies [6–8] and reflect poor adherence to guidelines [2,5]. Actual testing rates may be even lower than reported due to social desirability bias. HCPs’ decision to test for VVC likely depends on both provider-related and patient-related factors (e.g., risk factors, failed empiric treatment, recurrent or severe infections), which were not included in this survey. Perhaps unsurprisingly, OB/GYNs more frequently ordered diagnostic testing than other provider types, likely because of greater familiarity with guidelines for diagnosis, a focus on gynecologic health issues, and more routine use of pelvic examinations [7]. The higher rates of diagnostic testing in the Midwest are consistent with a previous claims-based study [7], but further research is needed to elucidate the reasons behind these regional differences in practice patterns. In addition, a better understanding of ways to increase proper diagnosis of VVC is needed, along with greater public health messaging to HCPs about the importance of diagnostic testing.

HCPs’ high reported rates of patient response to initial treatment are consistent with treatment success rates of 80–95% described elsewhere for topical or systemic azole antifungal medications [9]. However, other studies show that nearly half of patients with VVC receive inappropriate empiric treatment [7], raising the question whether resolution of symptoms reported in this survey was truly related to VVC diagnosis and treatment. The lack of association between diagnostic frequency and VVC outcomes is therefore likely highly confounded by misdiagnosis and clinician ascertainment and recall. Although we were not able to perform a multivariable analysis of factors associated with treatment success, bivariate suggests that more experienced providers with lower patient volume were more likely to report higher initial treatment success, possibly indicating the importance of allowing adequate time for each patient visit, tempered by indications that responses about treatment success may be biased and unreliable.

Our study’s main limitation is that the survey respondents may not be representative of the entire HCP population given the modest response rate. We also did not ask about specific diagnostic test types, follow up visits, or microbiological cure. More research to rigorously assess the benefits and drawbacks of more intensive diagnostics are needed, as well as the impact of misdiagnosis on patient health, healthcare costs, and antifungal resistance [10].
